# Supplementary material for: The genetic evolution of acral melanoma
Source: Nat Commun. 2024 Jul 21;15:6146. doi: 10.1038/s41467-024-50233-z (PMC11271482; doi:10.1038/s41467-024-50233-z)
Supplement: Supplementary file 1 — Supplementary Information [file 41467_2024_50233_MOESM1_ESM.pdf]

Supplementary Information

The genetic evolution of acral melanoma

Authors: Meng Wang, Satoshi Fukushima, Yi-Shuan Sheen, Egle Ramelyte, Noel Cruz Pacheco, Chenxu Shi, Shanshan Liu, Ishani Banik, Jamie D. Aquino, Martin Sangueza Acosta, Mitchell Levesque, Reinhard Dummer, Jau-Yu Liau, Chia-Yu Chu, A. Hunter Shain, Iwei Yeh, Boris C. Bastian

This document contains **Supplementary Figures 1-8**.

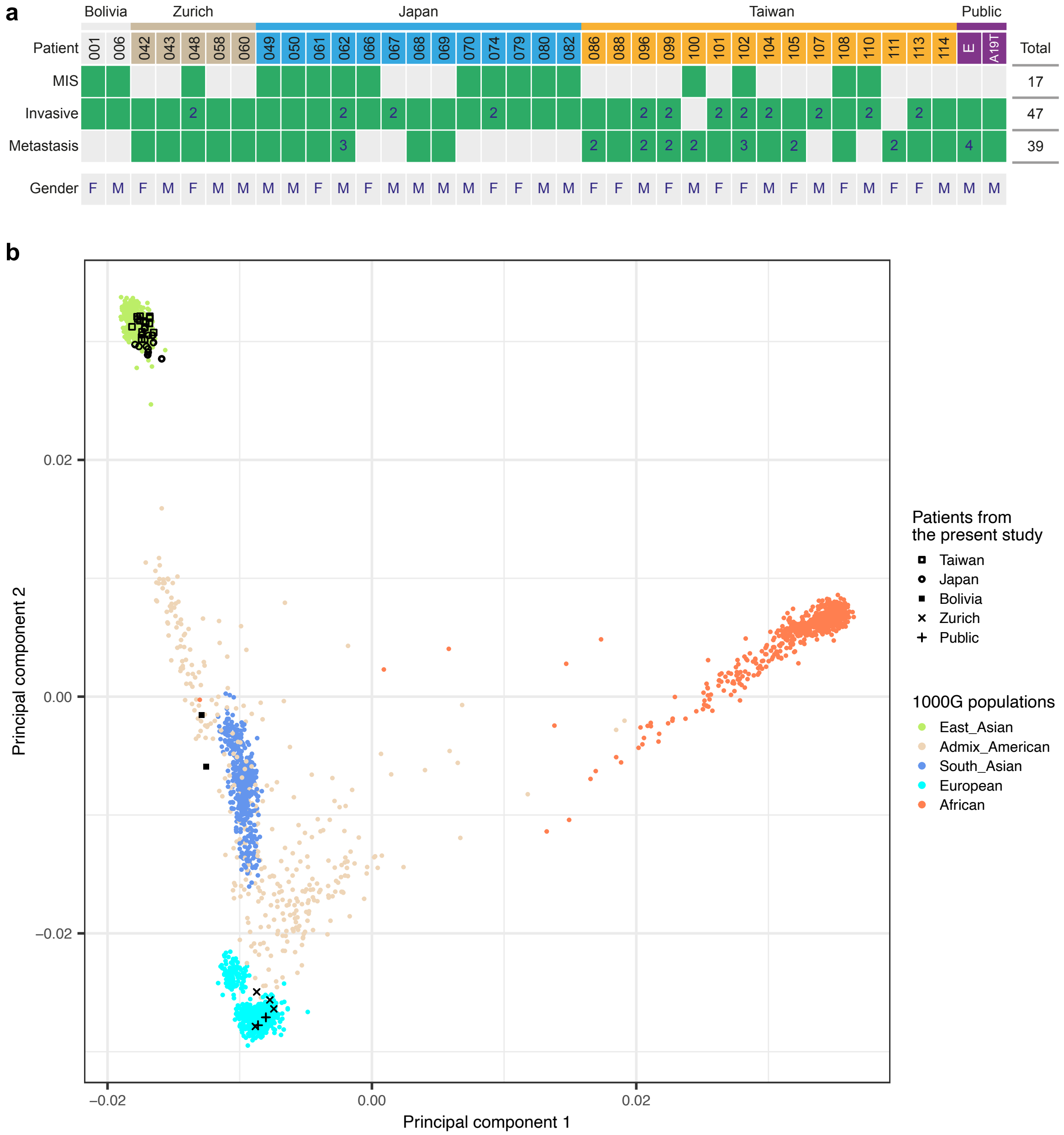

Supplementary Figure 1. Summary of all patients that were included in this study. a) Tumor samples (green colored) that were sequenced for each patient. If multiple samples were sequenced for a specific stage, the number is indicated. **b)** principal component analysis showing the ancestry/ethnicity of the patients. Case 042 could not be included due to the lack of a normal tissue sample. Source data are provided as a Source Data file.

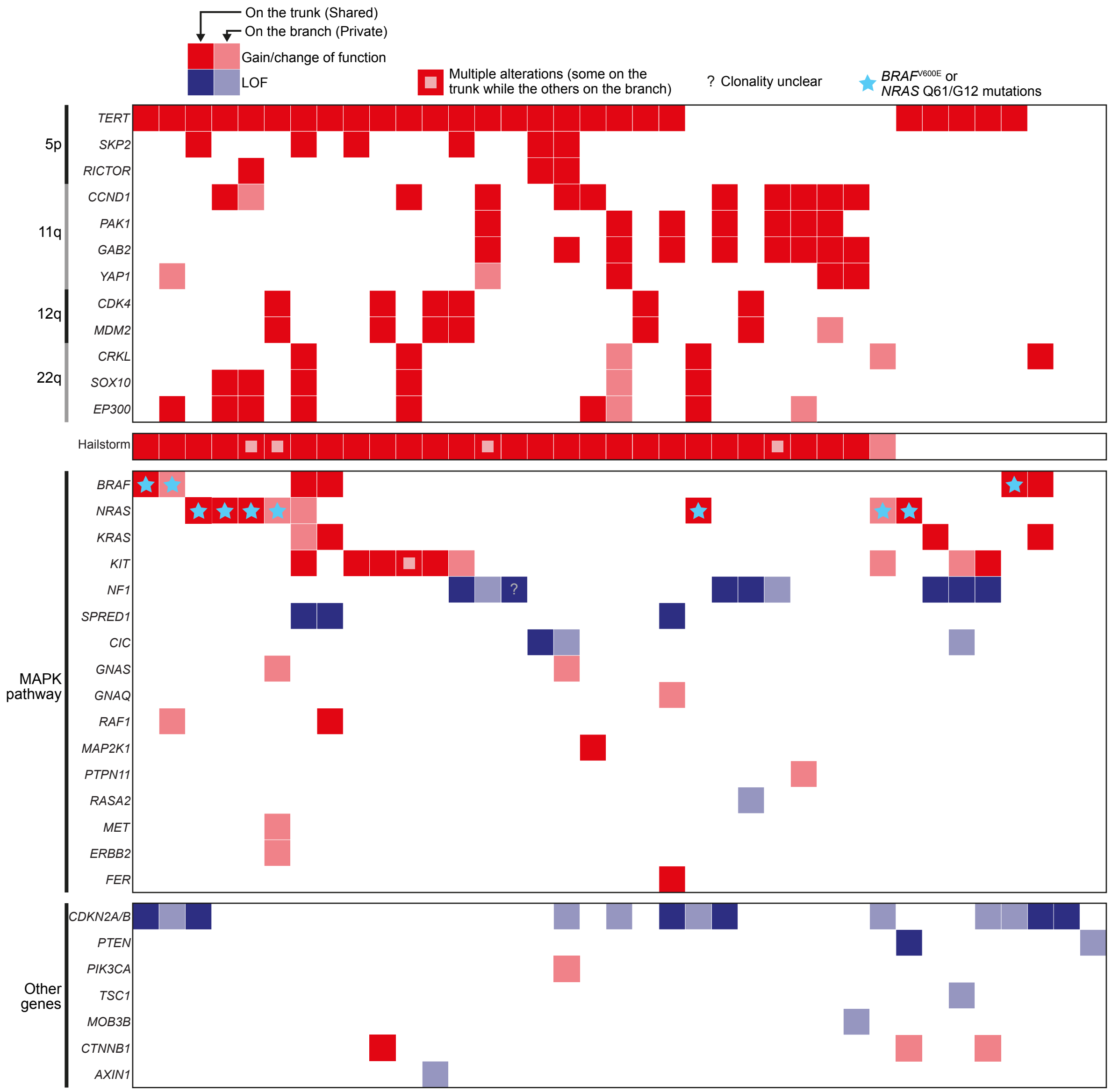

Supplementary Figure 2. Oncoplot showing the landscape of driver alterations. Source data are provided as a Source Data file.

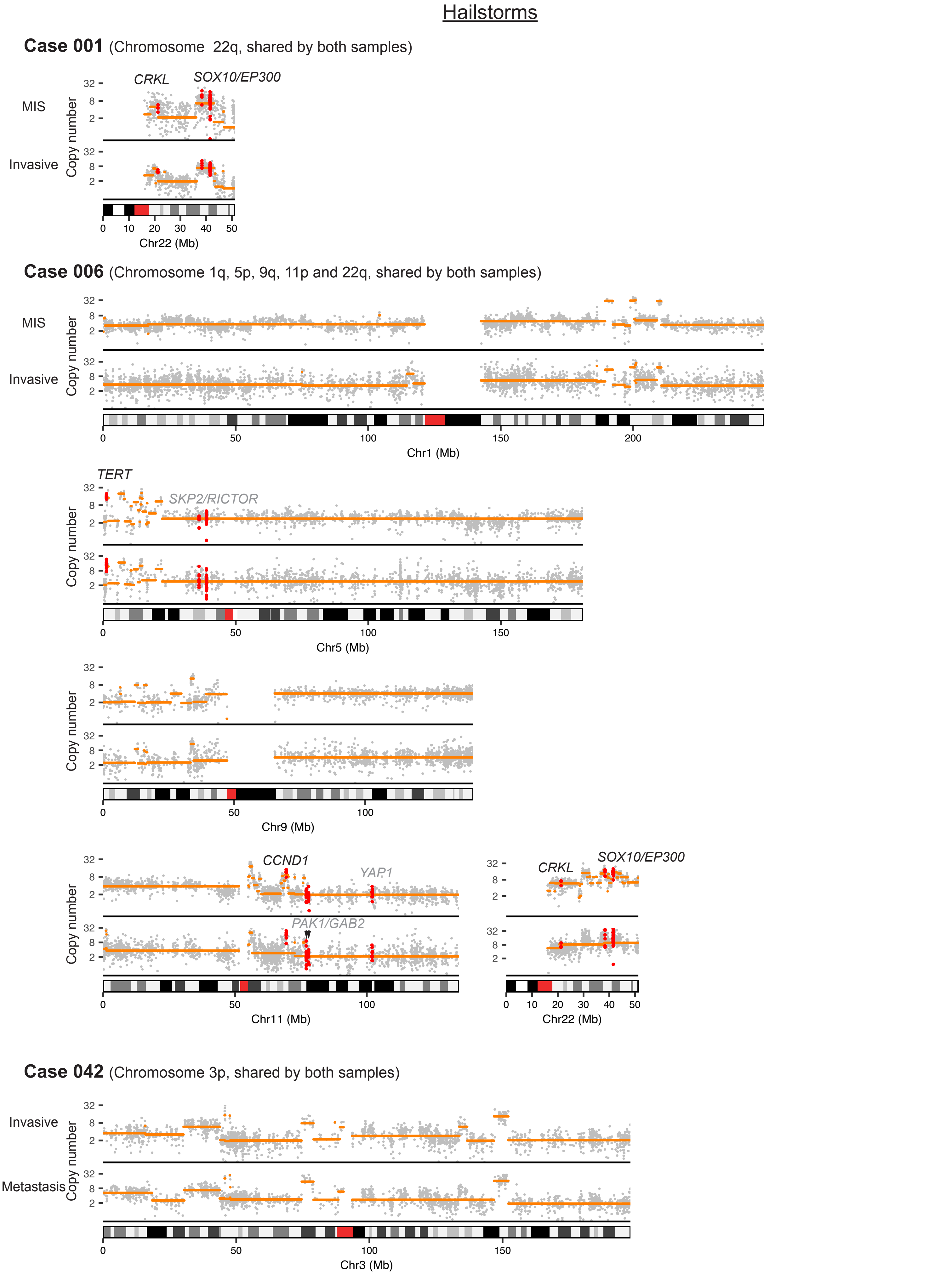

Note: CNVkit bins of selected genes are highlighted in red. The name of the genes are indicated, with amplified ones colored in black and unamplified ones in gray.

Supplementary Figure 3. Chromosomes with complex aberrations including hailstorms.

Case 049 (Chromosome 5p and 12q, shared by all samples; 7q, private to metastasis)

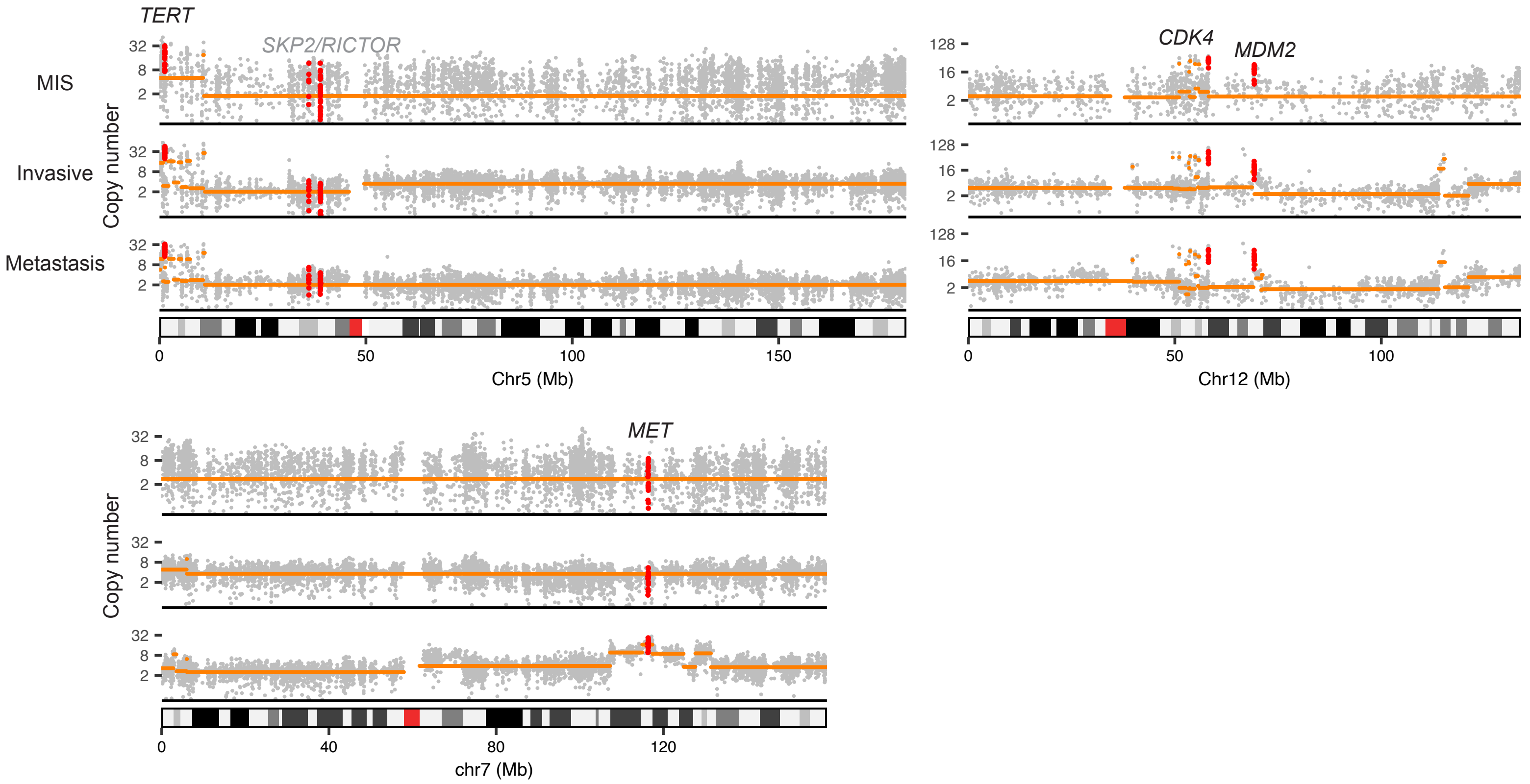

Case 050 (Chromosome 5p, 6q, 8q, 12q and Xq, shared by all samples)

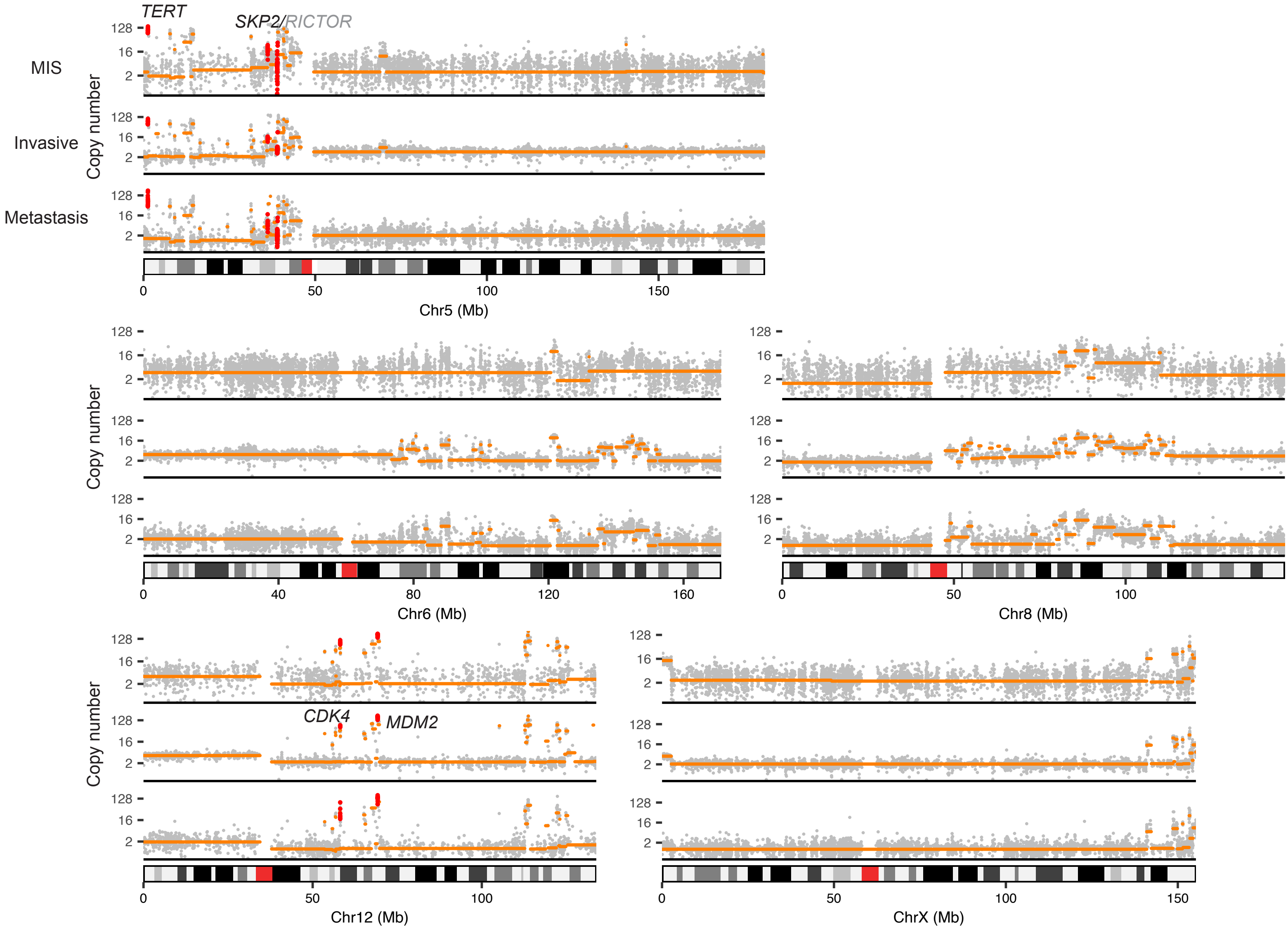

Case 058 (Chromosome 11q, private to metastasis; 22q, shared by both samples)

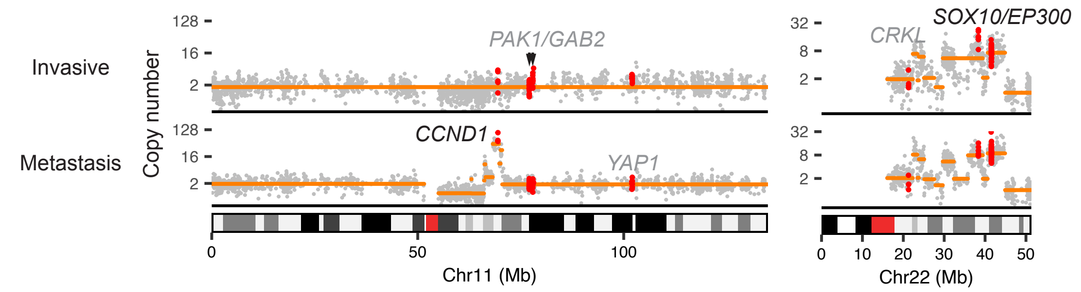

Case 060 (Chromosome 1p, shared by both samples)

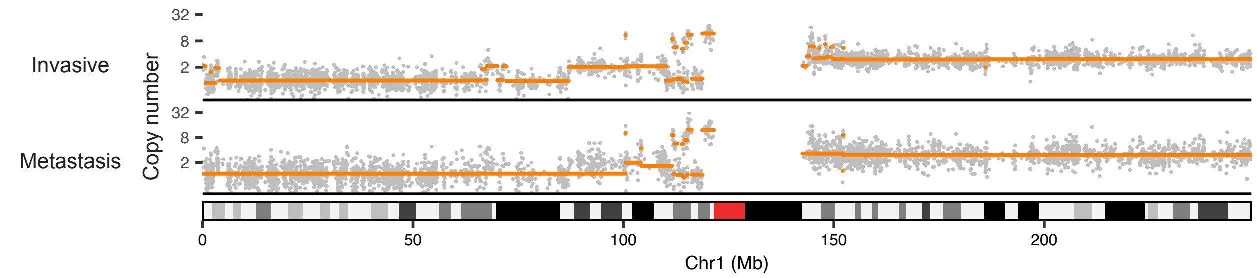

Case 061 (Chromosome 15q, private to MIS)

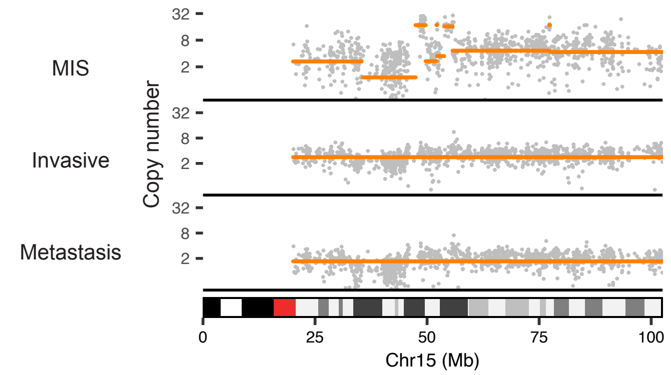

Case 062 (Chromosome 22q, shared by all samples)

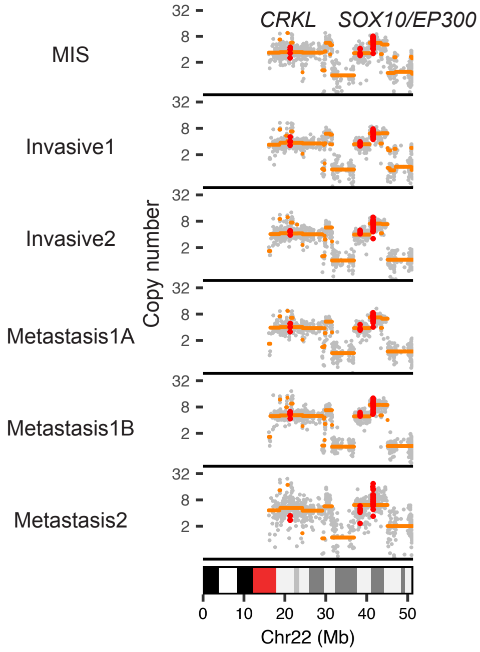

Case 067 (Chromosome 12q, shared by both samples)

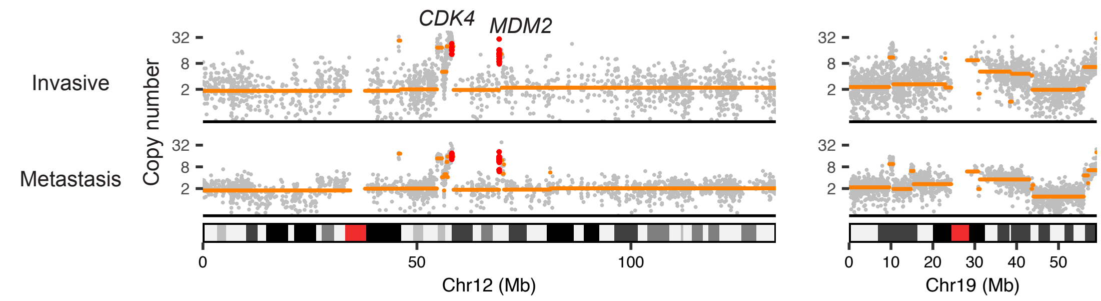

Case 068 (Chromosome 12q and 19q, shared by both samples)

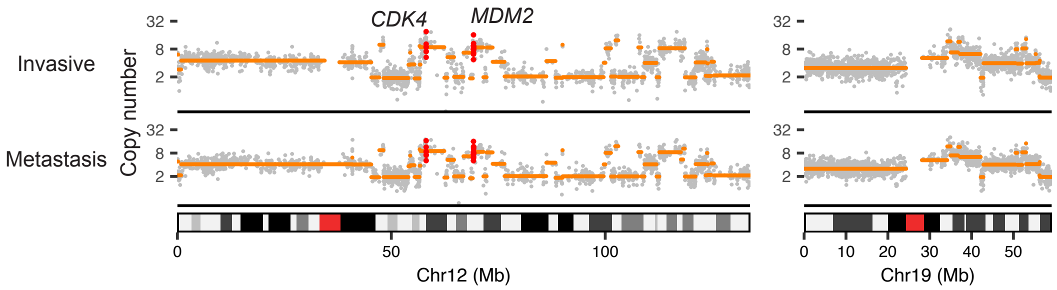

Case 070 (Chromosome 5p and 12p, shared by both samples)

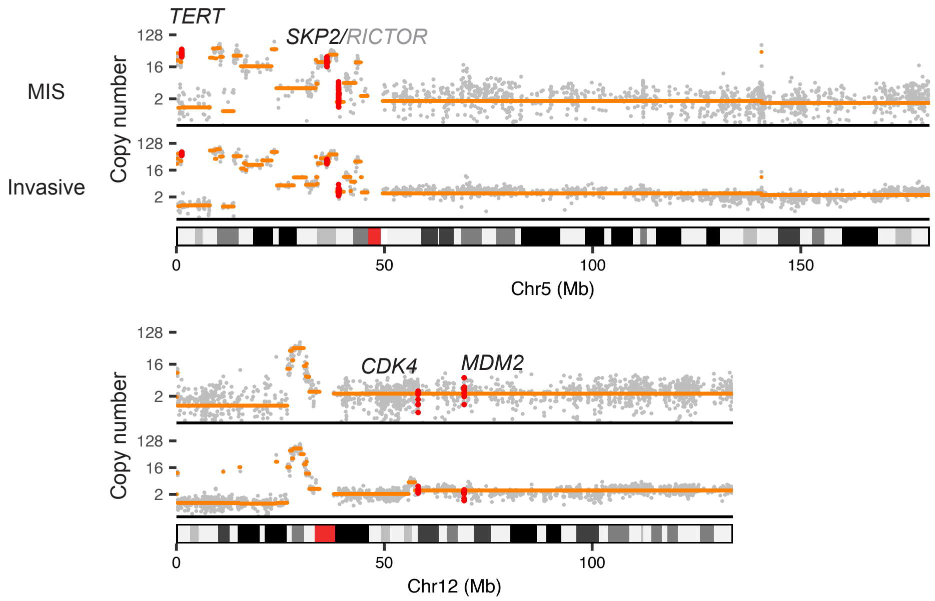

Case 074 (Chromosome 11q, shared by all samples; for 12q the amplified segments are narrow and considered as a simple focal amplicon)

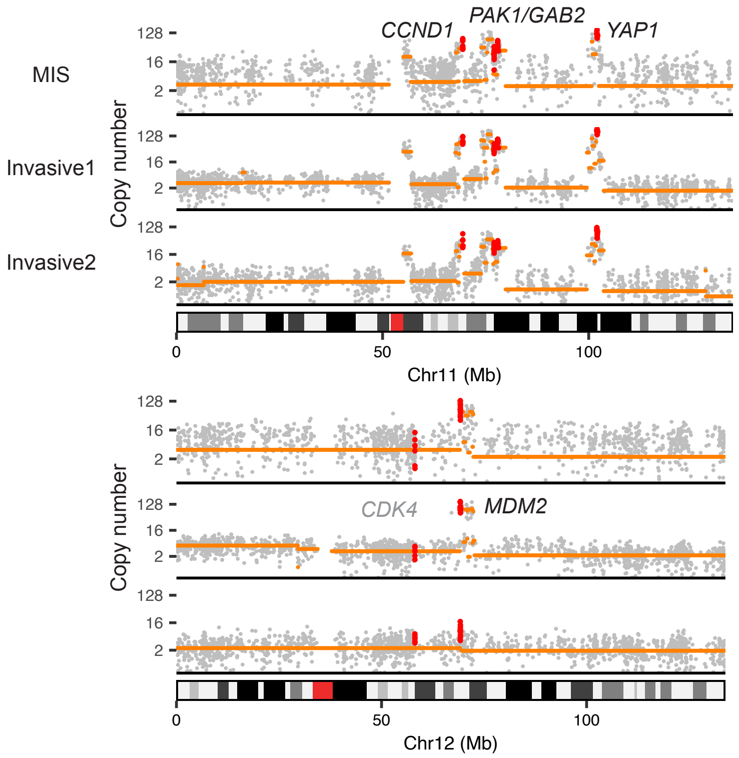

Case 079 (Chromosome 5q and 17q, likely shared by both samples. MIS has low purity)

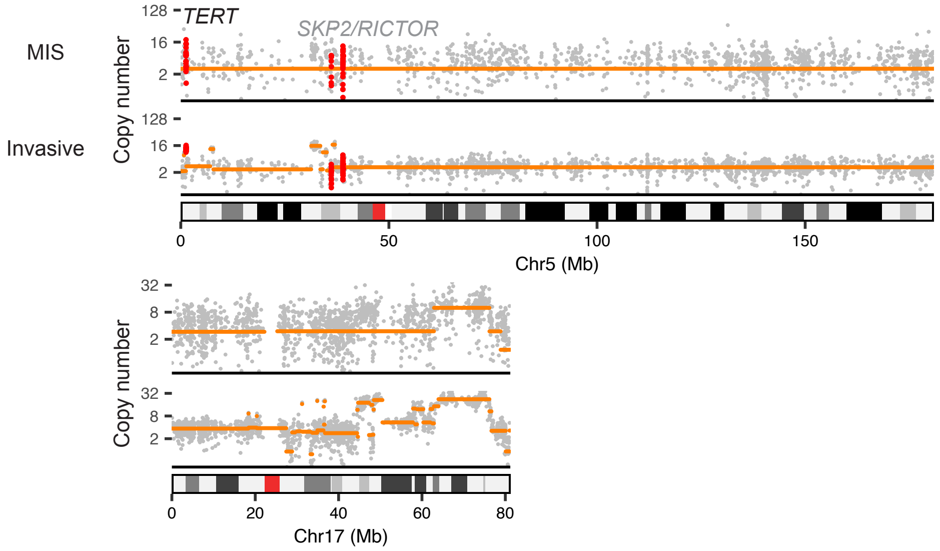

Case 080 (Chromosome 11q, shared by both samples.)

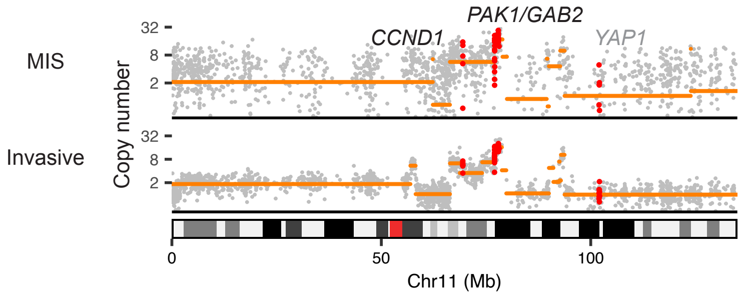

Case 082 (Chromosome 11q and 22q, likely shared by both samples. MIS has low purity)

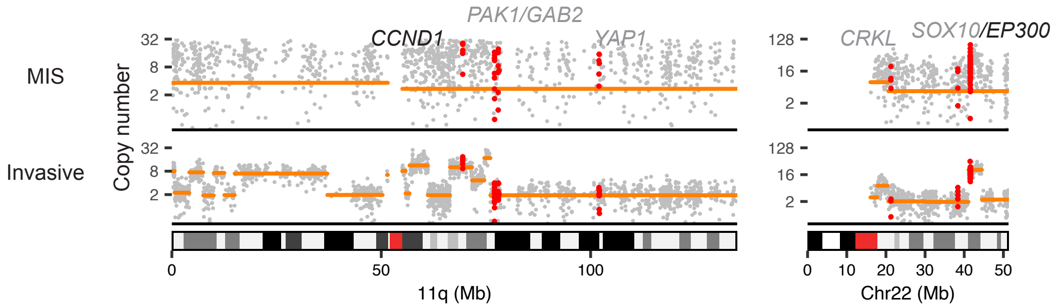

Case 086 (Chromosome 5p and 22q, shared by all samples)

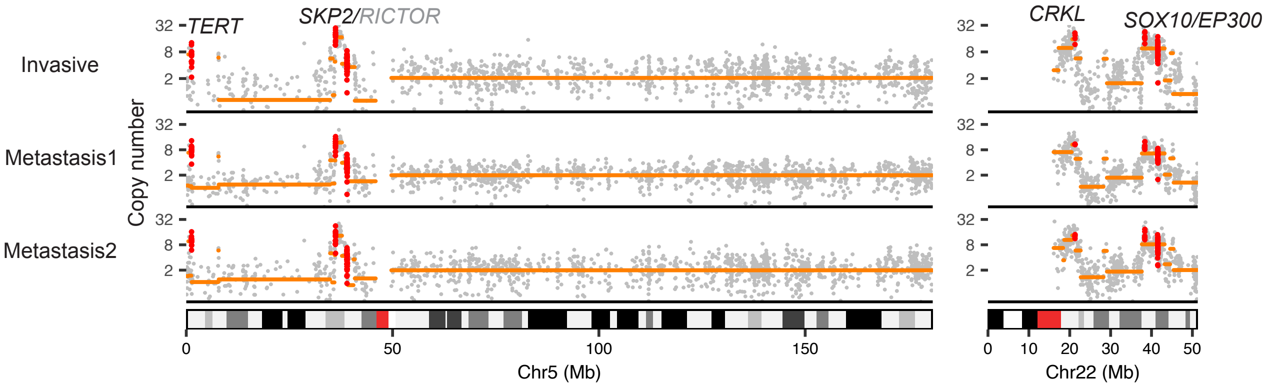

Supplementary Figure 3 cont.

Case 088 (Chromosome 3p, 5p and 7q, shared by both samples)

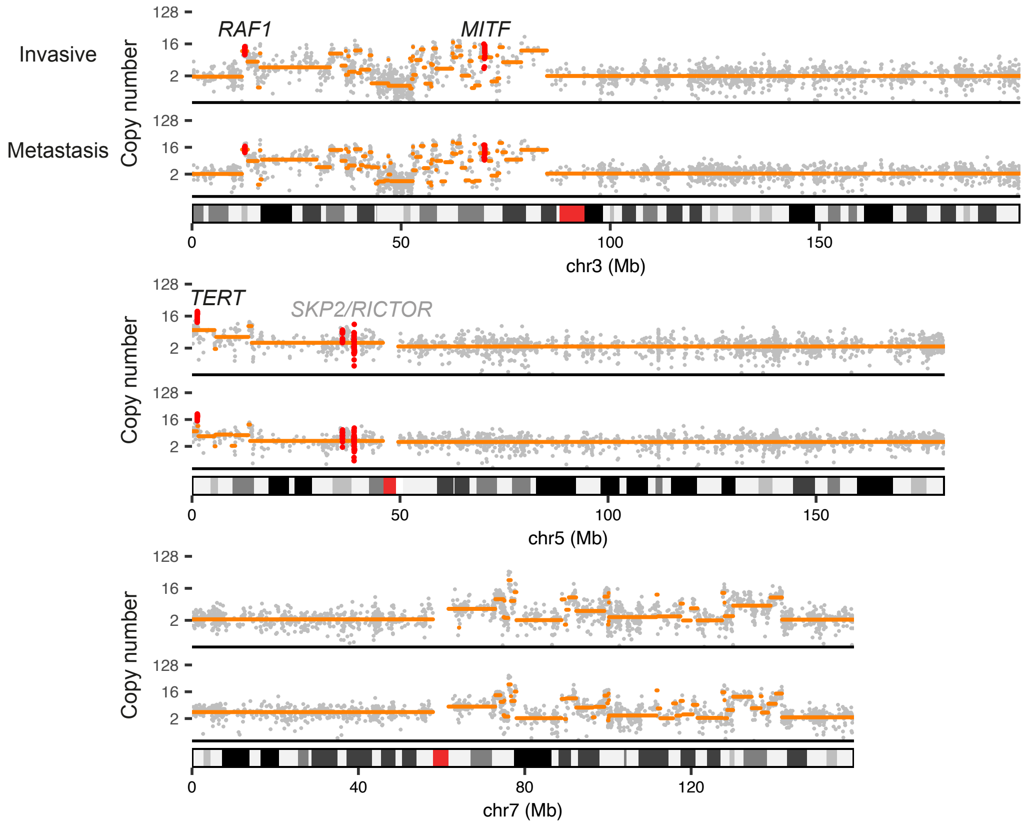

Case 096 (Chromosome 11q and 22q, shared by all samples)

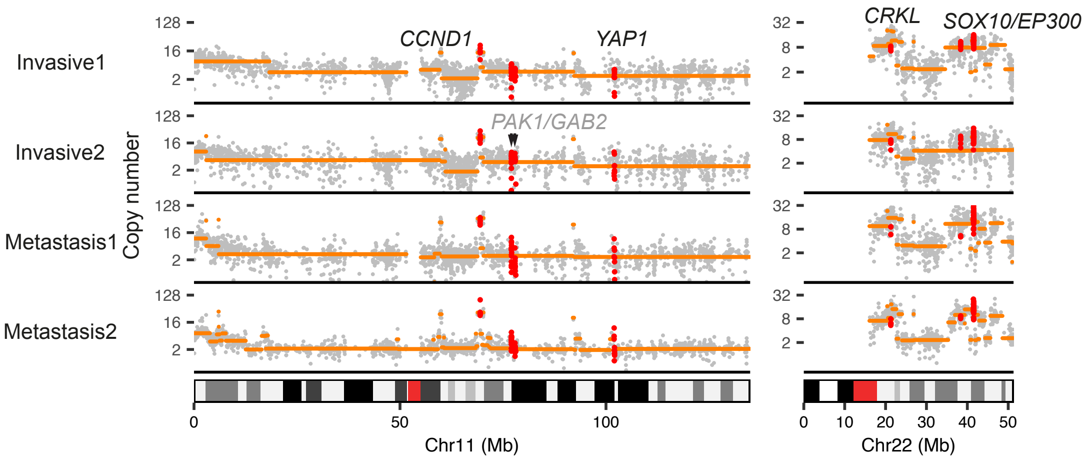

Case 100 (Chromosome 6q, 7q, 8q and 11q, shared by all samples; 2q, private to MIS and metastasis1)

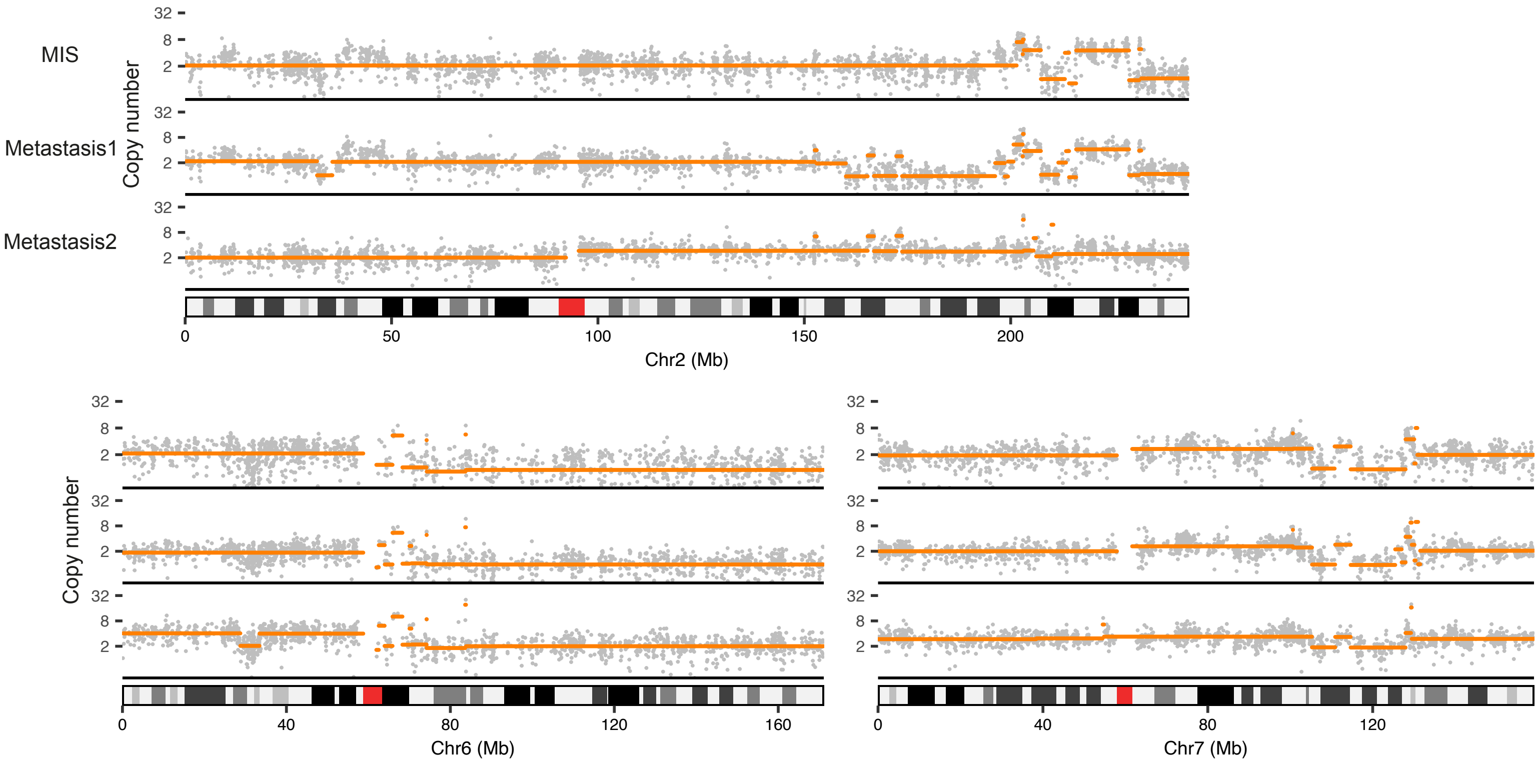

Case 100 (cont.)

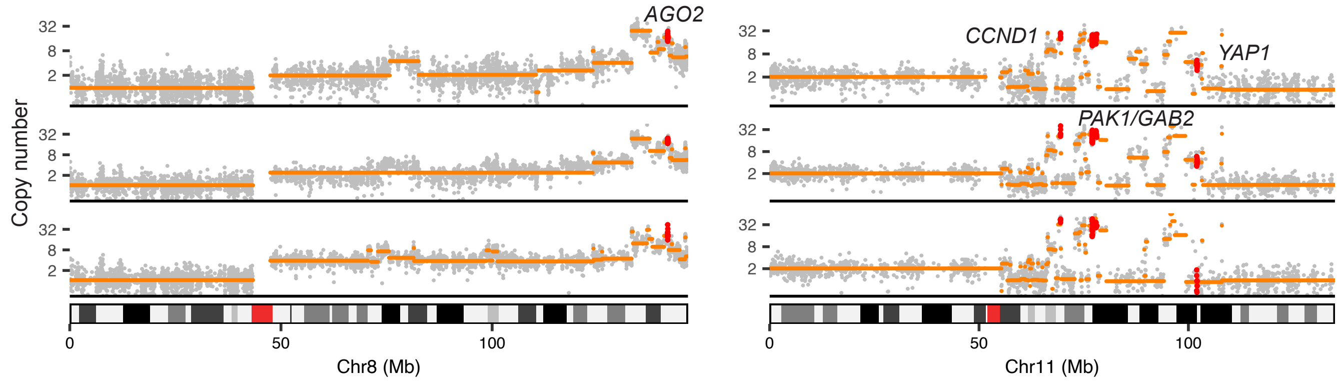

Case 101 (Chromosome 5p and 12q, shared by all samples)

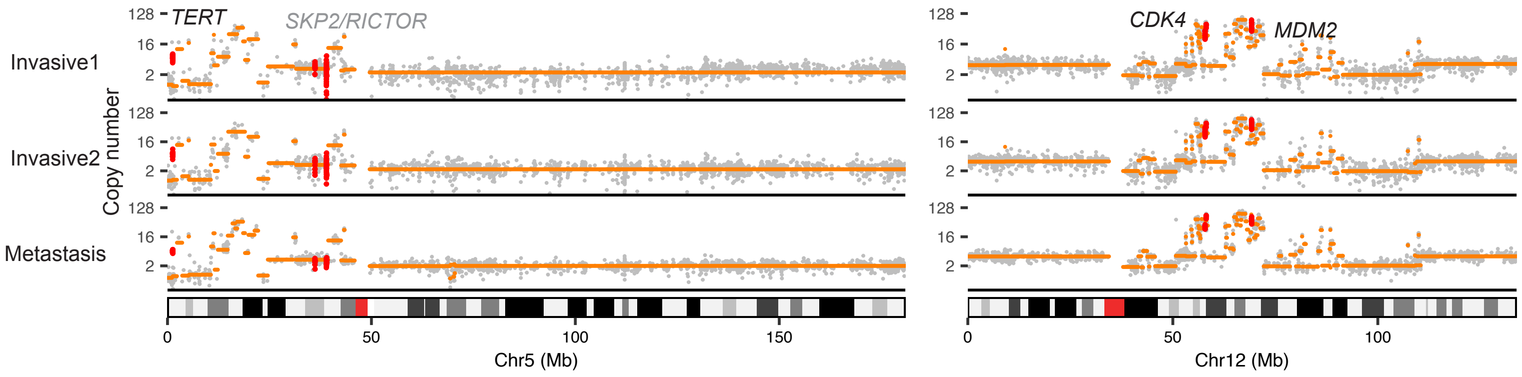

Case 102 (Chromosome 11q and 22q, shared by all samples)

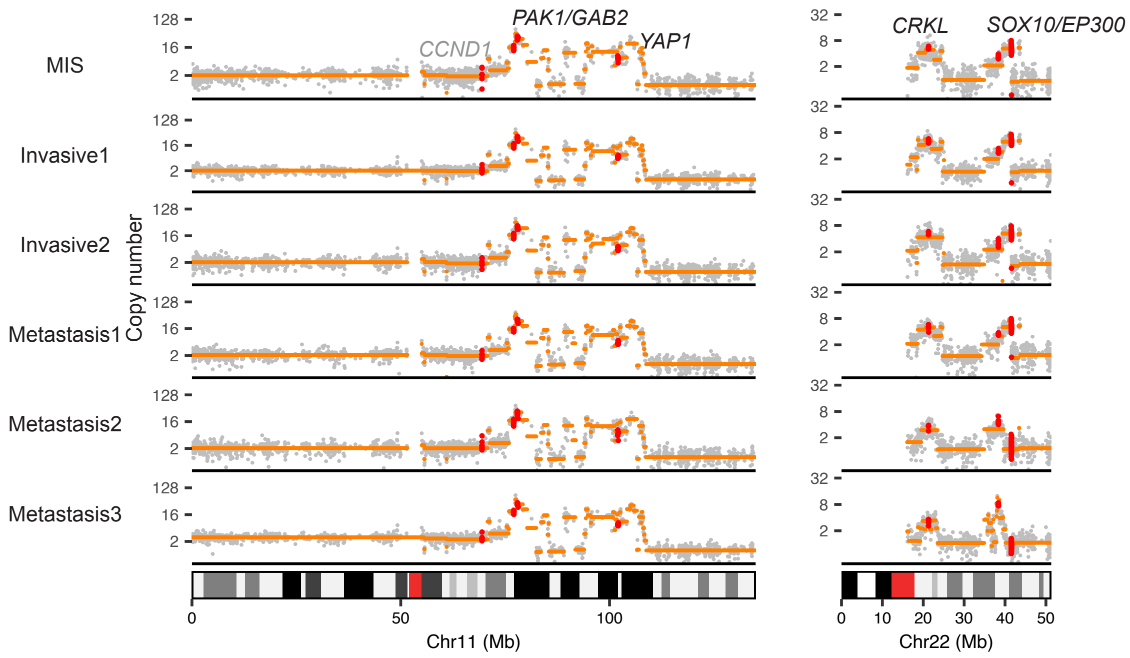

Case 104 (Chromosome 5p and 6q, shared by all samples)

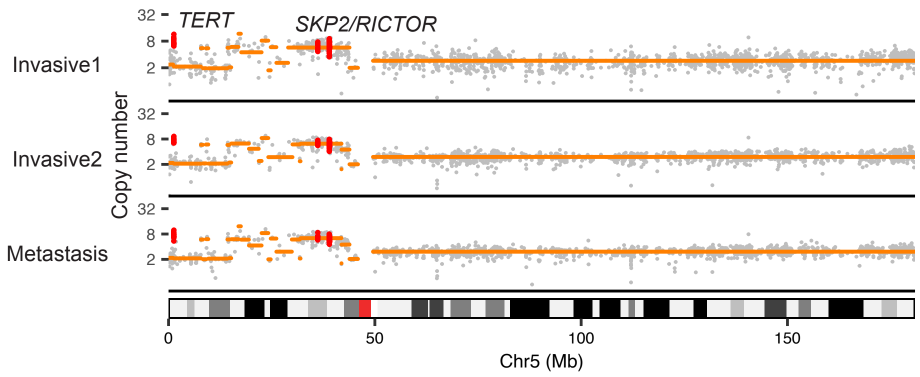

Case 104 (cont.)

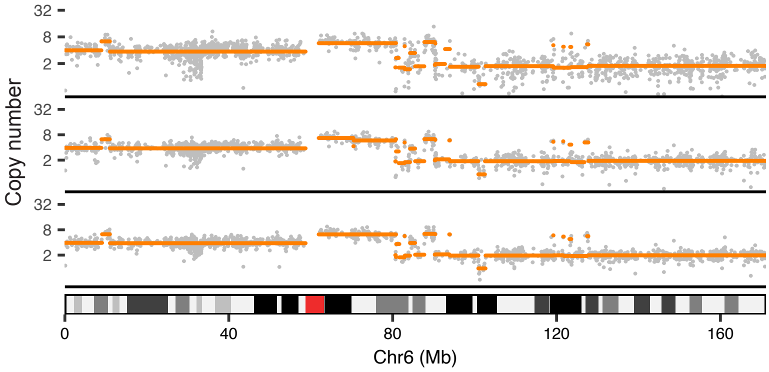

Case 105 (Chromosome 11q, shared by all samples)

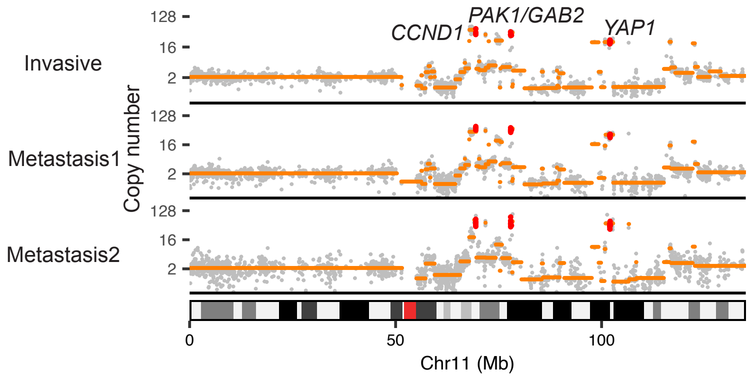

Case 110 (Chromosome 5p, 11q and 19q, shared by all samples)

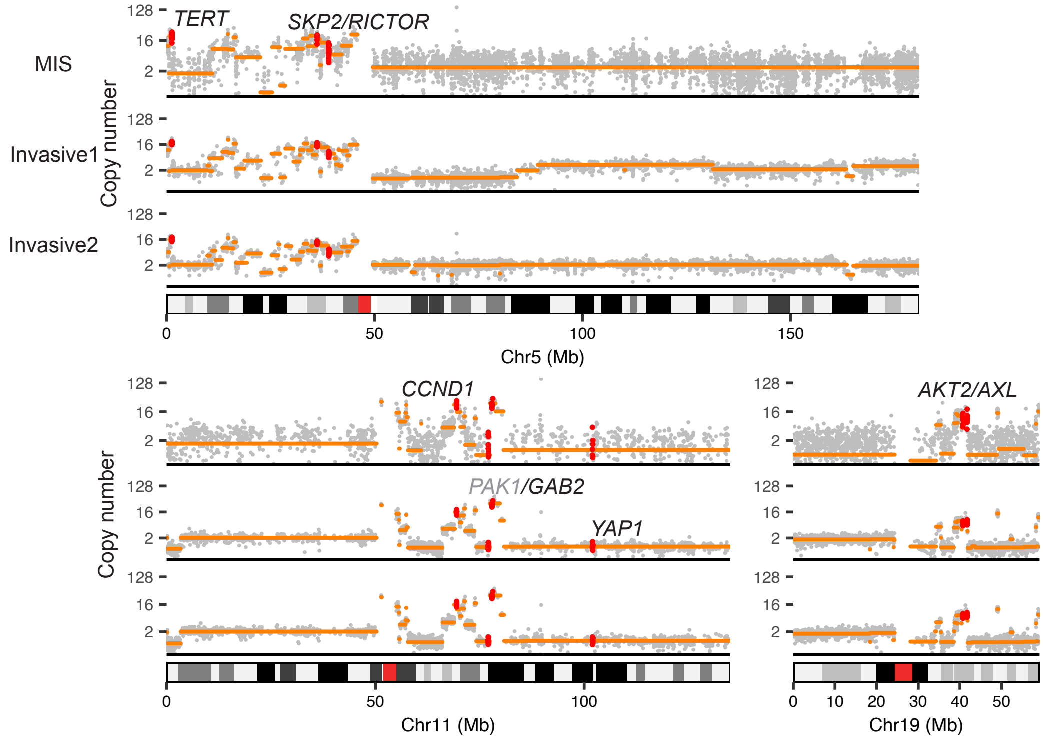

Case 111 (Chromosome 5p and 12q, shared by both samples)

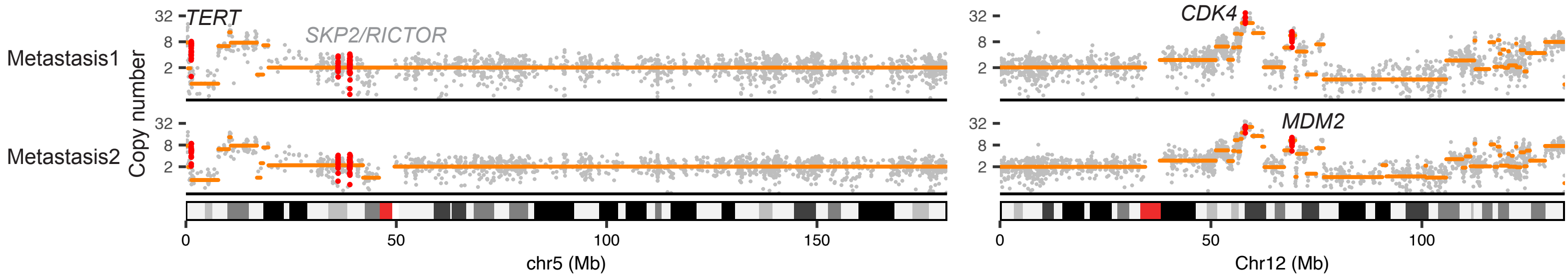

Case 113 (Chromosome 8q and 13q, shared by all samples)

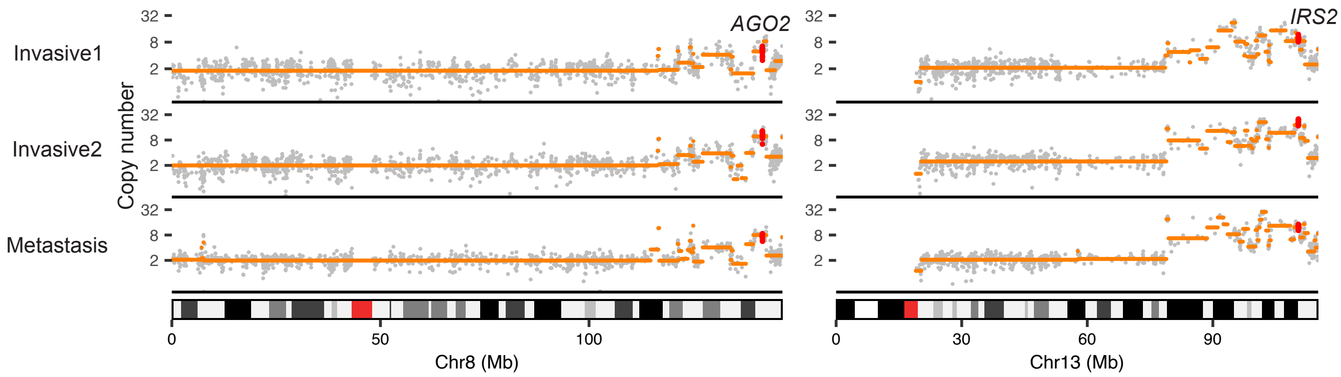

Case 114 (Chromosome 11q, shared by both samples)

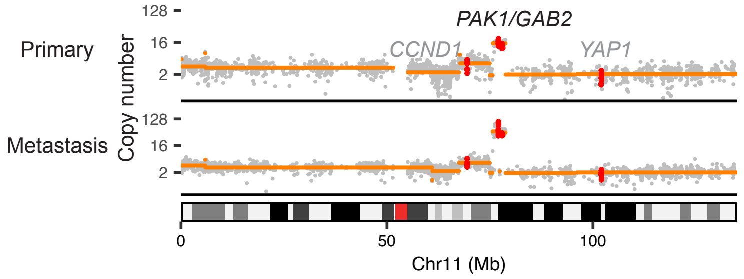

Case A19T (Chromosome 8q,11q and 15q, shared by both samples; 18q, private to metastasis)

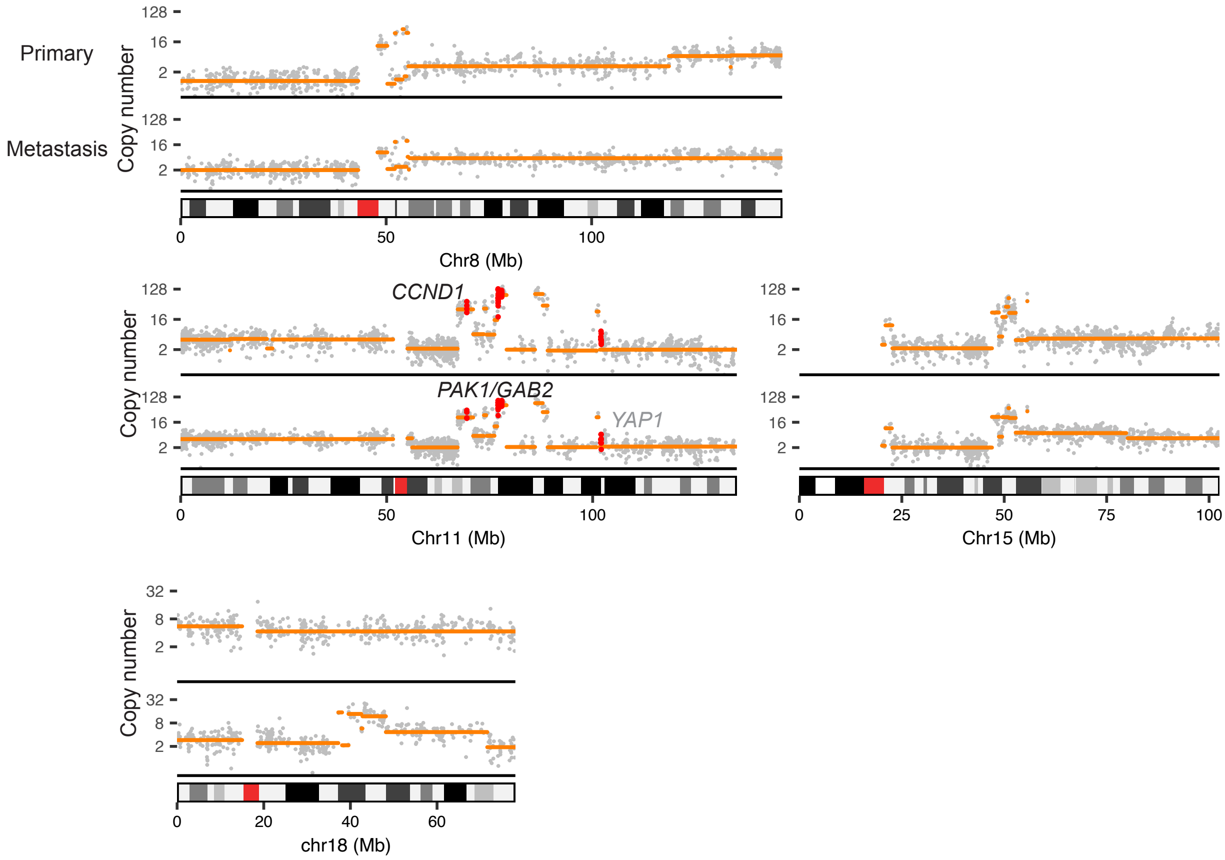

Additional chromothripsis-like aberrations

Case 048 (Chromosome 12q, shared by all samples)

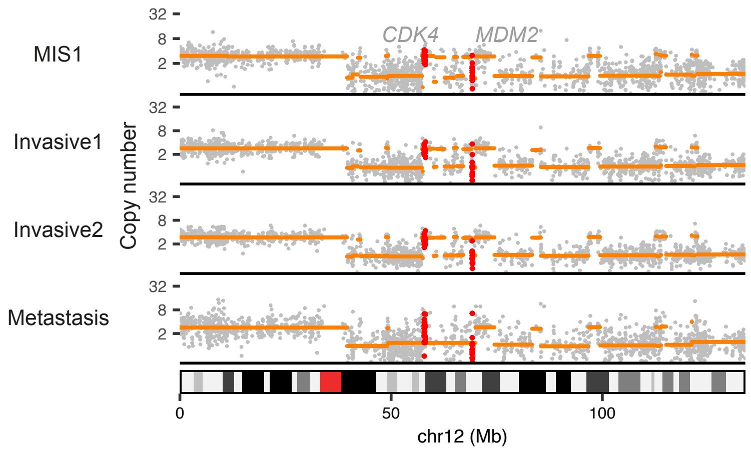

Case 099 (Chromosome 11q, shared by all samples)

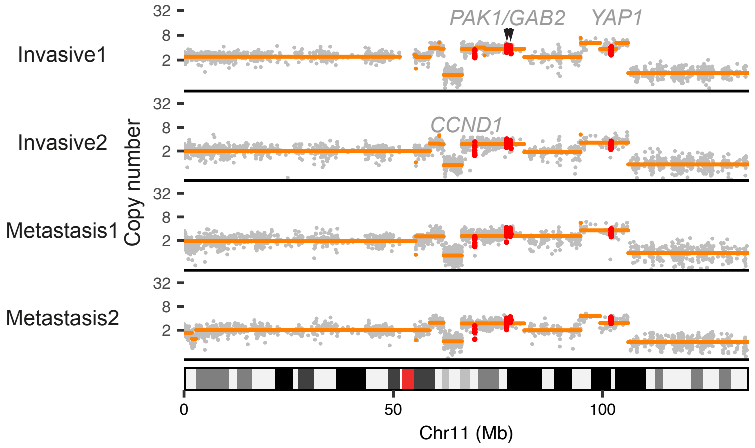

Case E (Chromosome 1q, shared by all samples)

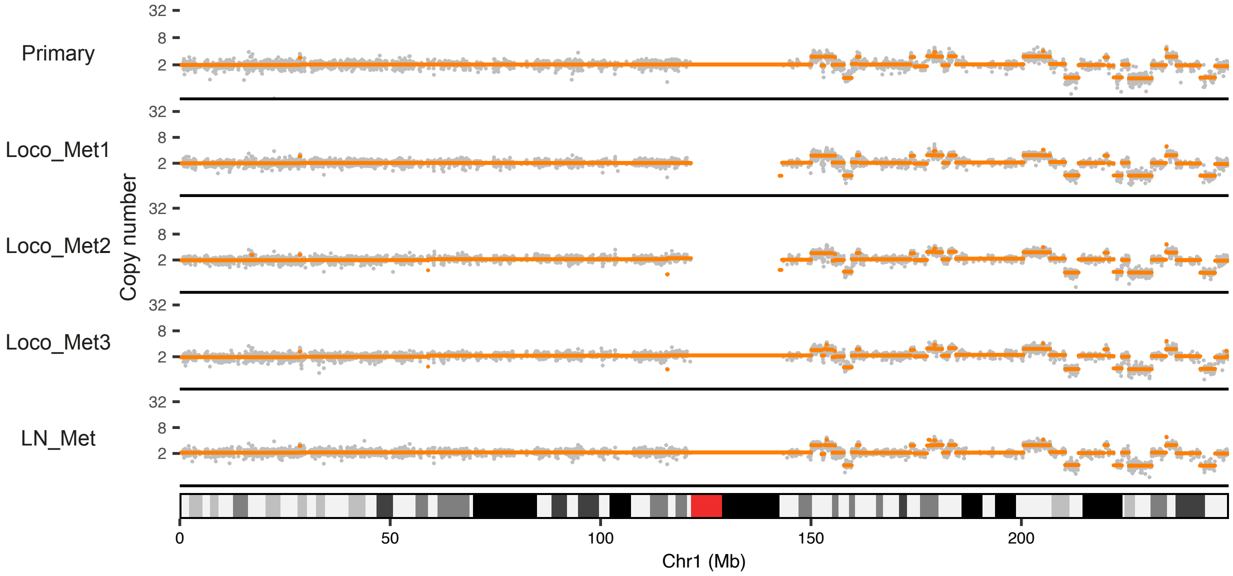

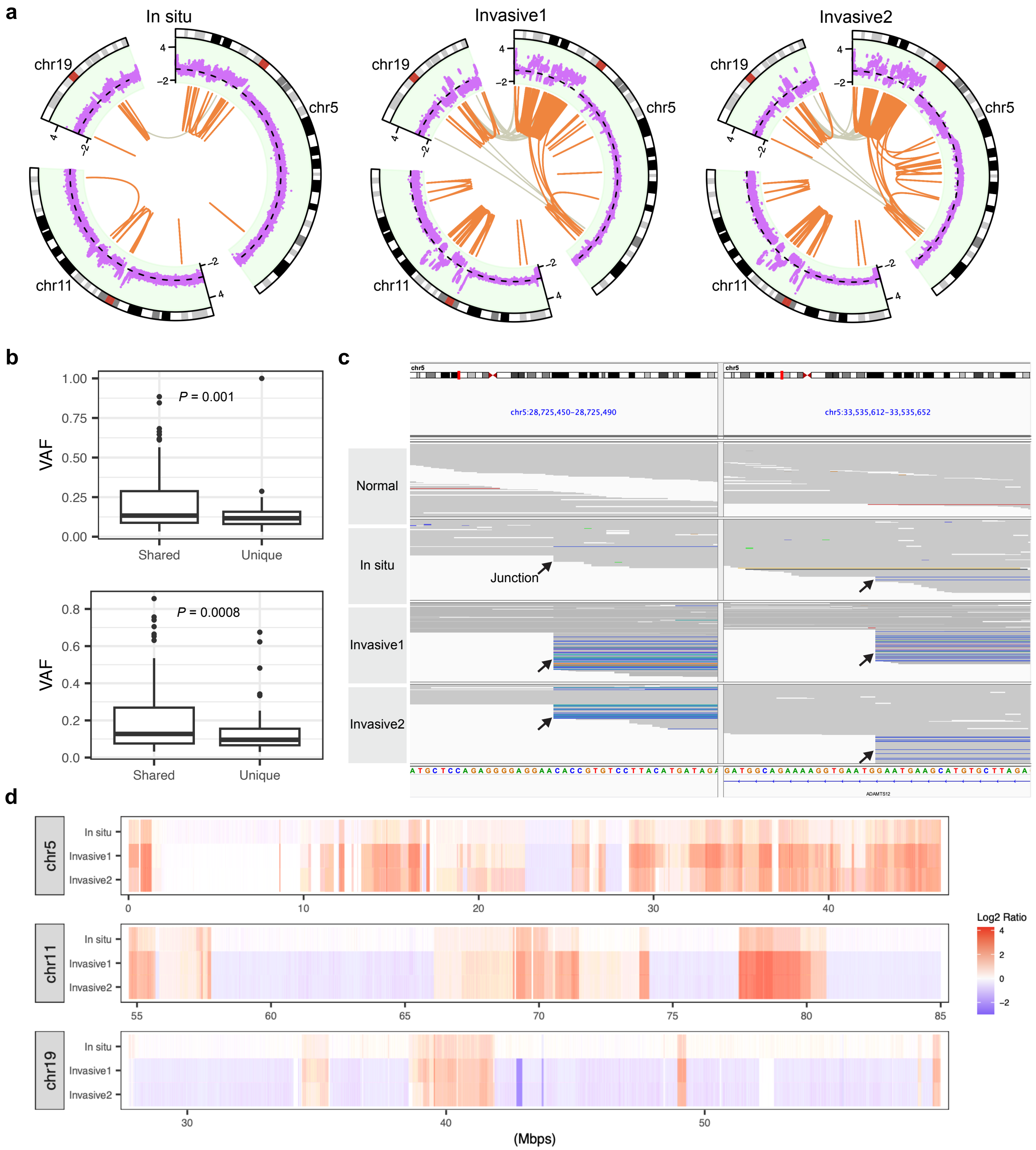

Supplementary Figure 4. Structural variation (SV) junctions and copy number profiles of hailstorms in case 110 (whole-genome sequencing data). a) Circos plots showing SV junctions on chromosomes 5, 11 and 19. Inter- and intra- chromosomal junctions are colored gray and orange, respectively. For invasive 1 and 2, we detected 286 and 317 SV junctions, respectively, across the genome. Approximately 72% (208/286 and 229/317) of the SVs in each involved 5p, 11q and 19q, with 150 common to both areas. In the *in situ* area, 27 junctions were detected on 5p, 11q or 19q, probably due to its lower tumor purity, with 22 (81.5%) also detected in the two invasive areas. Inter- chromosomal SVs were observed between chr5 and chr19. The outer track shows the chromosome ideograms; the middle track shows the log2 ratio of tumor to normal

copy number (purple dots) with the dashed black line indicating 0. **b)** The variant allele frequencies (VAFs) of junctions common to the two invasive areas were significantly higher than those confined to one area. Top: invasive 1; bottom: invasive 2. Medians (black line inside boxes), 25/75 percentiles (upper and lower boundaries of boxes), maximum/minimum (whiskers) and outliers (dots) were shown. **c)** Manual inspection identified shared SVs that were initially missed during detection in the *in situ* area, as illustrated by the example SV. **d)** Heatmaps based on whole-genome sequencing data show highly concordant copy number profiles on 5p, 11q and 19q. Source data are provided as a Source Data file.

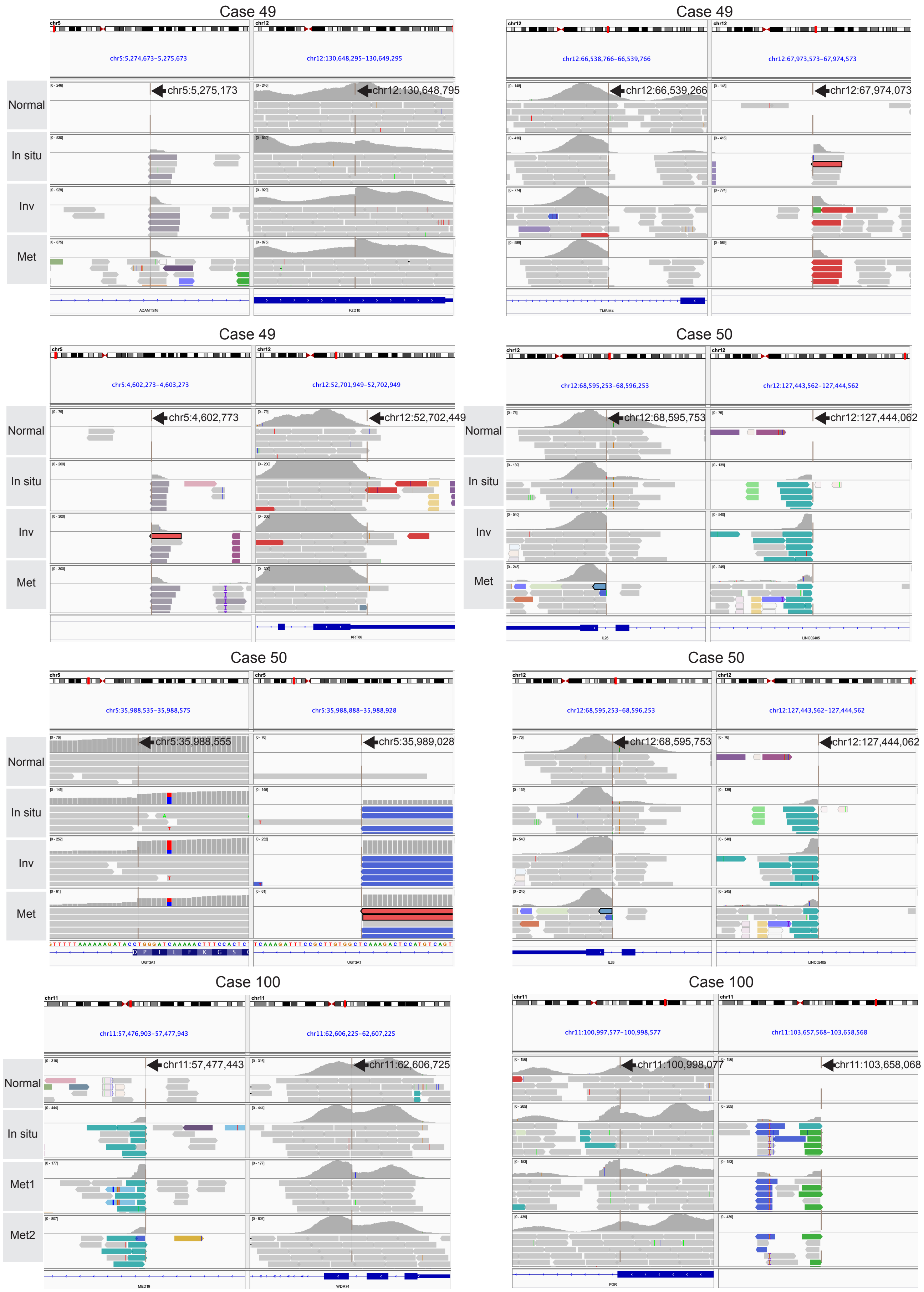

Supplementary Figure 5. Truncal SV junctions within hailstorms detected by whole-exome sequencing data. The dashed lines in each panel signify the breakpoints of SV junctions, with corresponding genomic coordinates.

Supplementary Figure 5 cont.

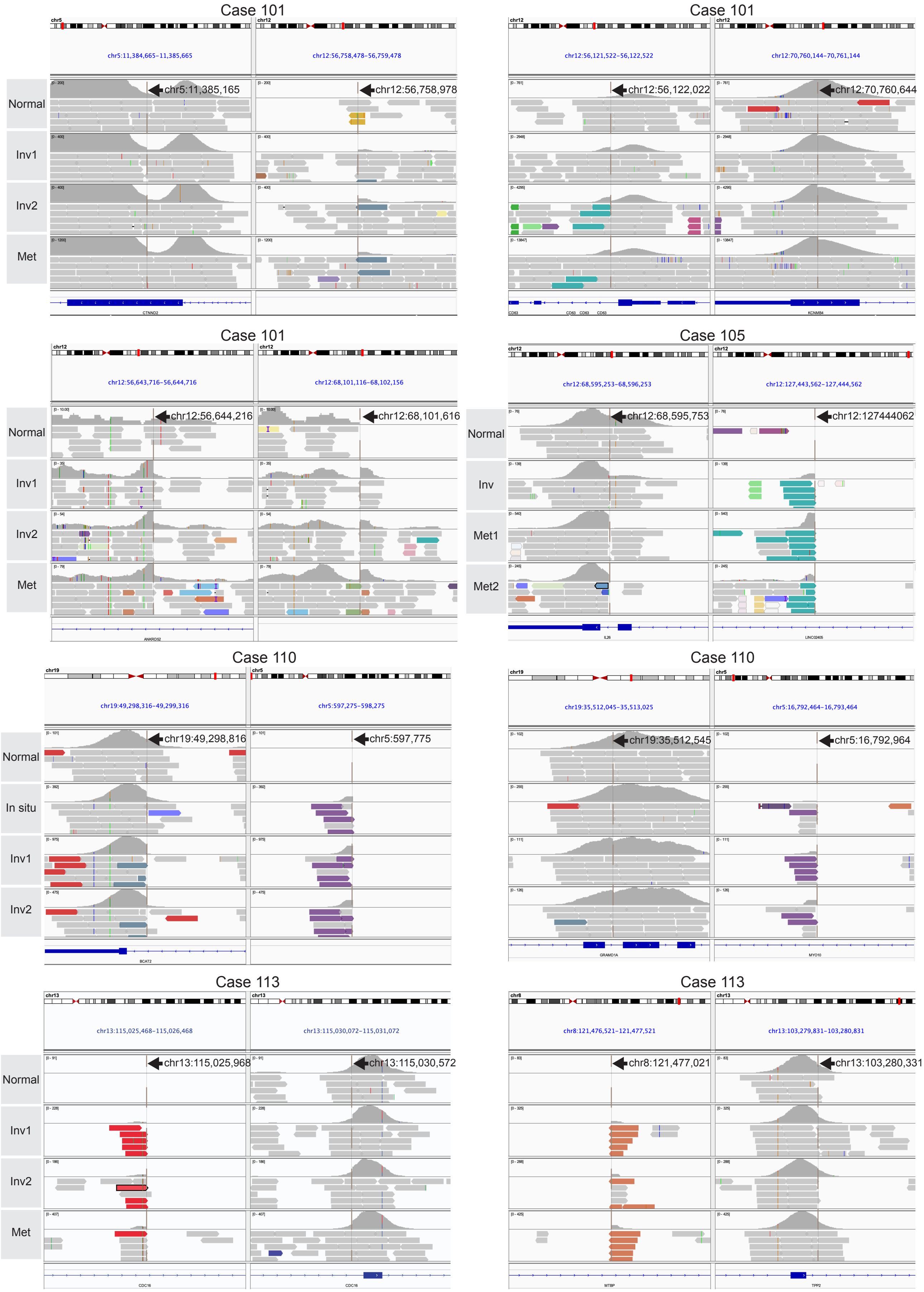

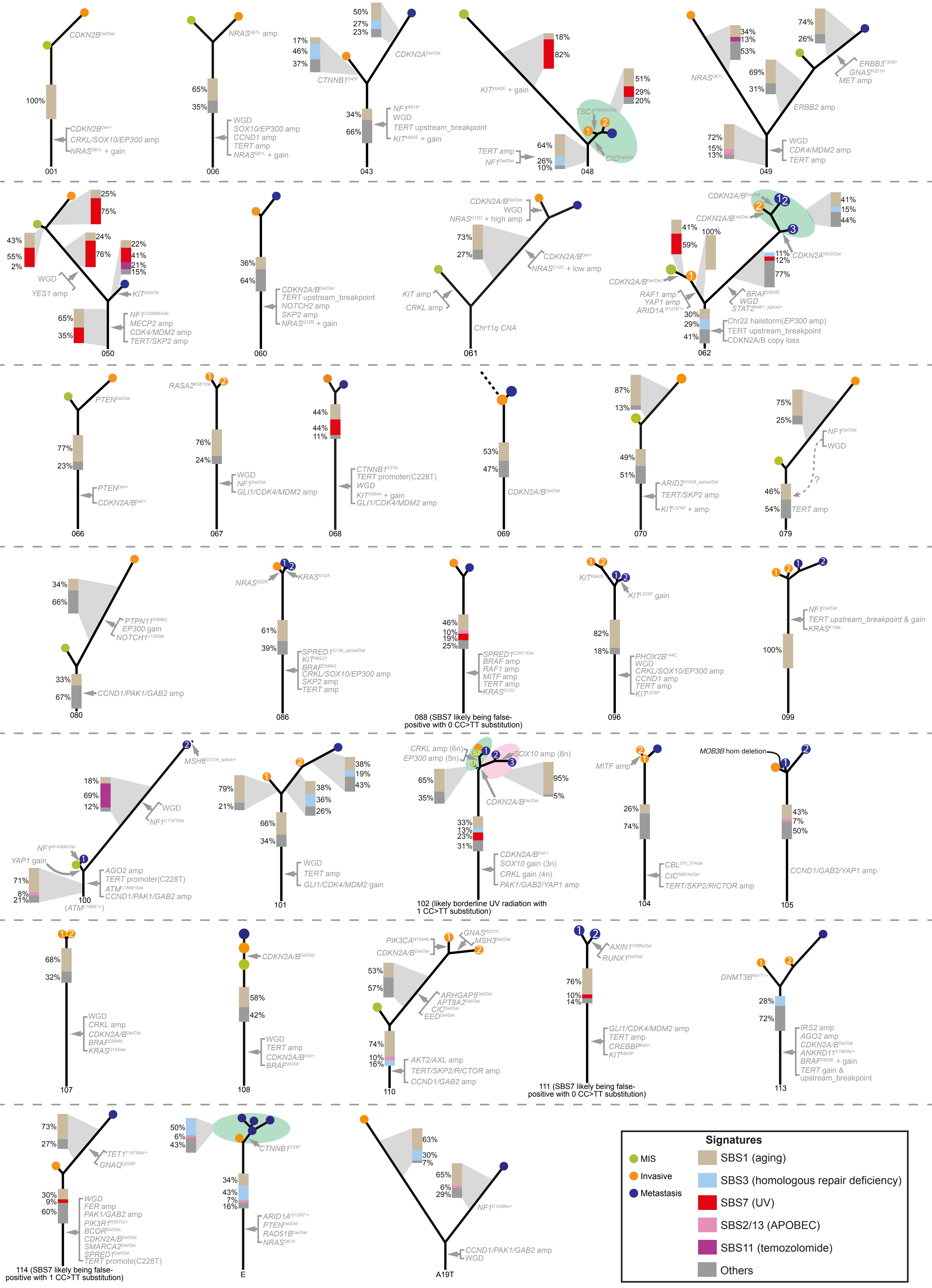

001
006
043
048
049
050
060
061
062
066
067
068
069
070
079
080
086
088 (SBS7 likely being false-positive with 0 CC>TT substitution)
096
099
100
101
102 (likely borderline UV radiation with 1 CC>TT substitution)
104
105
107
108
110
111 (SBS7 likely being false-positive with 0 CC>TT substitution)
113
114 (SBS7 likely being false-positive with 1 CC>TT substitution)
E
A19T
Signatures
MIS
Invasive
Metastasis
SBS1 (aging)
SBS3 (homologous repair deficiency)
SBS7 (UV)
SBS2/13 (APOBEC)
SBS11 (temozolomide)
Others

Supplementary Figure 6. Mutation signatures and their intratumor distribution. Cases with sufficient somatic mutations to reliably call mutational signatures are shown.

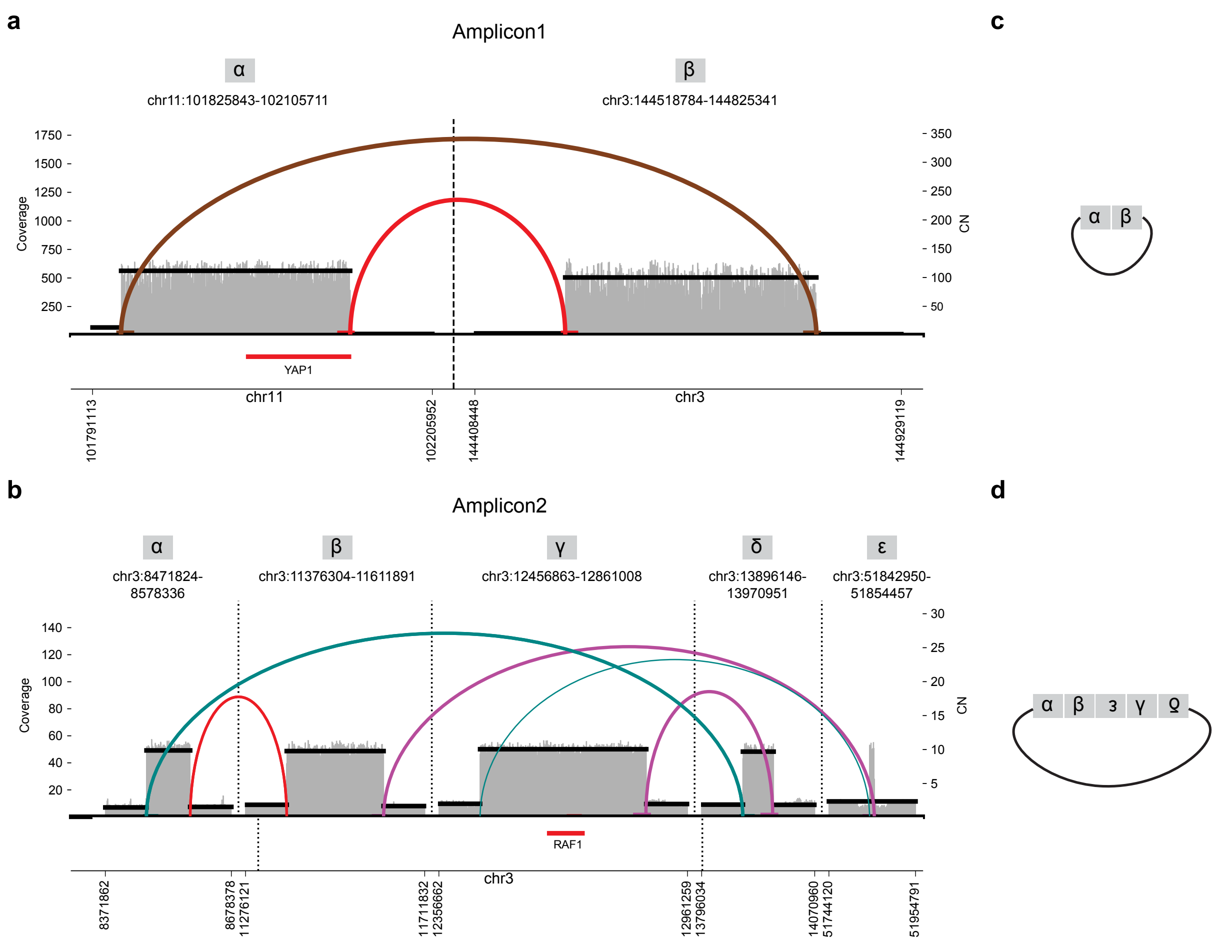

Supplementary Figure 7. Two probable extrachromosomal DNAs (ecDNAs) revealed by AmpliconArchitect. **a** and **c**) The detailed architecture of the two amplicons, with SV junctions, as denoted by arched lines, indicating circular structures for both. The amplified segments, with genomic coordinates indicated, are labelled with Greek letters on top of each panel. **b** and **d**) Diagrams illustrating the order and orientation of the amplified segments.

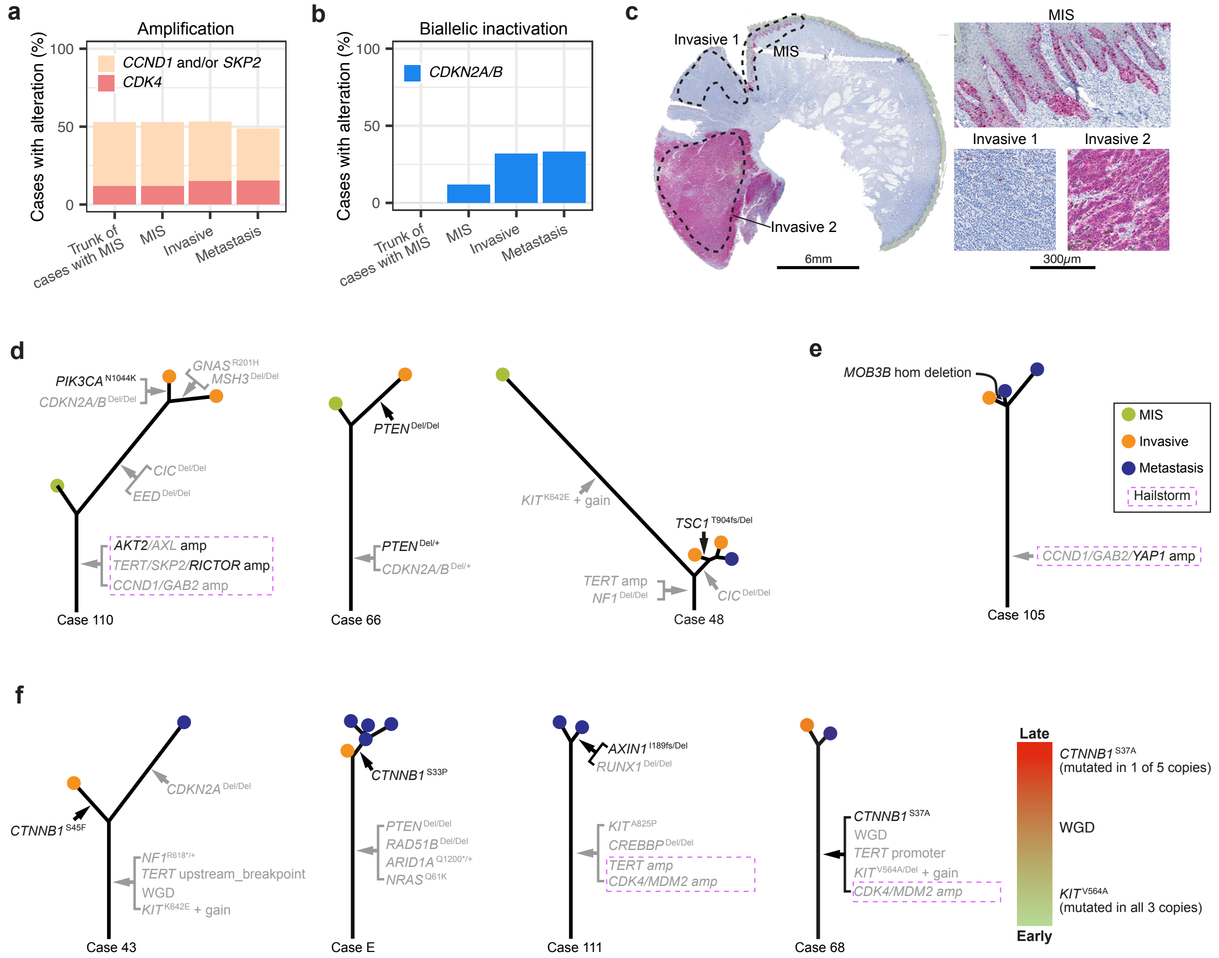

Supplementary Figure 8. Sequential order of genetic alterations of the G1/S cell cycle checkpoint and other important pathways. a) Amplifications of *CCND1*, *SKP2* and *CDK4* mostly occurred at the earliest detectable stage and were maintained during progression. **b)** Biallelic inactivation of *CDKN2A/B* mostly occurred at the transition to invasive melanoma. **c)** Heterogeneity of *CDKN2A/B* alteration in case 110. *CDKN2A/B* was homozygously deleted in invasive area 1, but not the flanking MIS and invasive area 2. The immunohistochemistry of p16 confirms loss of expression in invasive area 1. Scale bars are indicated in panel. **d)** Three cases with alterations of *PIK3CA*, *PTEN* and *TSC1* from the PI3K/AKT/MTOR signaling pathway that arose later during progression. **e)** One case with a late emerging *MOB3B* alteration in addition to a truncal *YAP1* amplification. **f)** Cases with mutations of *CTNNB1* or *AXIN1* from the WNT pathway emerging later during progression. For case 68, *CTNNB1* ^{S37A} was situated on the trunk, but likely arose after *KIT* ^{V564A} and WGD. Source data are provided as a Source Data file.
