# Supplementary material for: The genetic evolution of acral melanoma
Source: Nat Commun. 2024 Jul 21;15:6146. doi: 10.1038/s41467-024-50233-z (PMC11271482; doi:10.1038/s41467-024-50233-z)
Supplement: Supplementary file 3 — Description of Additional Supplementary Files [file 41467_2024_50233_MOESM3_ESM.pdf]

Description of Additional Supplementary Files

Supplementary Data 1. Clinical information and sequencing metrics of all sequencing areas.

Supplementary Data 2. List of somatic mutations for all tumor samples.

Supplementary Data 3. List of copy number segments for all tumor samples.

Supplementary Data 4. The bait regions of a panel of 80 cancer genes.
