# Supplementary material for: The genetic evolution of acral melanoma
Source: Nat Commun. 2024 Jul 21;15:6146. doi: 10.1038/s41467-024-50233-z (PMC11271482; doi:10.1038/s41467-024-50233-z)
Supplement: Supplementary file 8 — Reporting Summary [file 41467_2024_50233_MOESM8_ESM.pdf]

nature portfolio

Corresponding author(s): Boris C. Bastian

Last updated by author(s): 06/27/2024

Reporting Summary

Nature Portfolio wishes to improve the reproducibility of the work that we publish. This form provides structure for consistency and transparency in reporting. For further information on Nature Portfolio policies, see our Editorial Policies and the Editorial Policy Checklist.

Statistics

For all statistical analyses, confirm that the following items are present in the figure legend, table legend, main text, or Methods section.

n/a	Confirmed	
☐	☒	The exact sample size (*n*) for each experimental group/condition, given as a discrete number and unit of measurement
☐	☒	A statement on whether measurements were taken from distinct samples or whether the same sample was measured repeatedly
☐	☒	The statistical test(s) used AND whether they are one- or two-sided<br>*Only common tests should be described solely by name; describe more complex techniques in the Methods section.*
☒	☐	A description of all covariates tested
☒	☐	A description of any assumptions or corrections, such as tests of normality and adjustment for multiple comparisons
☒	☐	A full description of the statistical parameters including central tendency (e.g. means) or other basic estimates (e.g. regression coefficient) AND variation (e.g. standard deviation) or associated estimates of uncertainty (e.g. confidence intervals)
☐	☒	For null hypothesis testing, the test statistic (e.g. *F*, *t*, *r*) with confidence intervals, effect sizes, degrees of freedom and *P* value noted<br>*Give P values as exact values whenever suitable.*
☒	☐	For Bayesian analysis, information on the choice of priors and Markov chain Monte Carlo settings
☒	☐	For hierarchical and complex designs, identification of the appropriate level for tests and full reporting of outcomes
☒	☐	Estimates of effect sizes (e.g. Cohen's *d*, Pearson's *r*), indicating how they were calculated

Our web collection on statistics for biologists contains articles on many of the points above.

Software and code

Policy information about availability of computer code

Data collection	No software was used for data collection.
Data analysis	FastQC v0.11.9<br>fastp v0.23.4<br>Burrows-Wheeler Aligner v0.7.17<br>Picard v2.19.0<br>Genome AnalysisTK v3.6.0, v4.1.2.0<br>freebayes v1.3.2<br>Strelka2 v2.9.10<br>ANNOVAR 2018Apr16<br>FACETS v0.6.2<br>Delly2 v0.8.7<br>CNVkit v0.9.6<br>AmpliconSuite-pipeline v1.1.1<br>SAMtools v1.10, v1.18<br>BCFtools v1.18<br>PLINK v1.90b6.24<br>ggplot2<br>R v4.1.0<br>Integrated Genome Browser v2.6.3

deconstructSigs v1.9.0
Custom codes: https://github.com/Bioinfowangm/Genetic-evolution-acral-melanoma

For manuscripts utilizing custom algorithms or software that are central to the research but not yet described in published literature, software must be made available to editors and reviewers. We strongly encourage code deposition in a community repository (e.g. GitHub). See the Nature Portfolio guidelines for submitting code & software for further information.

Data

Policy information about availability of data

All manuscripts must include a data availability statement. This statement should provide the following information, where applicable:

- Accession codes, unique identifiers, or web links for publicly available datasets
- A description of any restrictions on data availability
- For clinical datasets or third party data, please ensure that the statement adheres to our policy

The BAM format raw whole-exome, whole-genome and targeted sequencing data of all samples generated in this study have been deposited to the dbGaP database under accession number phs003451 [https://www.ncbi.nlm.nih.gov/projects/gap/cgi-bin/study.cgi?study_id=phs003451]. The sequencing data are available under restricted access for privacy consideration and regulatory compliance. Permanent employees of an institution at a level equivalent to a tenure-track professor or senior scientist with laboratory administration and oversight responsibilities may request access through dbGAP. The sequencing data of melanomas from the TCGA-SKCM project were downloaded from the NCI Genomics Data Commons, under dbGaP accession number phs000178 [https://www.ncbi.nlm.nih.gov/projects/gap/cgi-bin/study.cgi?study_id=phs000178.v11.p8]. The sequencing data of case E is available from dbGaP under accession number phs000941.v1.p1 [https://www.ncbi.nlm.nih.gov/projects/gap/cgi-bin/study.cgi?study_id=phs000941.v1.p1]. Summary of genetic findings of all 37 cases included in this study can be found at Figshare [https://doi.org/10.6084/M9.FIGSHARE.22773791]17. Source data are provided with this paper.

Research involving human participants, their data, or biological material

Policy information about studies with human participants or human data. See also policy information about sex, gender (identity/presentation), and sexual orientation and race, ethnicity and racism.

Reporting on sex and gender	This study does not involve the categorization of participants based on their sex or gender. However, the sex of each participant was reported as additional information.
Reporting on race, ethnicity, or other socially relevant groupings	This study does not involve the categorization of participants based on their race, ethnicity, or other socially relevant groupings.
Population characteristics	History of past treatment is considered related to tumor mutation signature and therefore analyzed. Other characteristics were not considered as relevant.
Recruitment	Participants were included if at least two out of the three acral melanoma progression stages (in situ, invasive and metastasis) were available.
Ethics oversight	This study was approved by the Institutional Review Board at the University of California, San Francisco (Parnassus Committee), and regional Ethical Committees at Kumamoto University (the Ethics Committee for Human Genome and Gene Analysis Research, Graduate School of Life Sciences), National Taiwan University Hospital (Research Ethics Committee C), University of Zurich (Kantonale Ethikkommission Zurich), and Hospital Obrero (Research Ethics Committee of Hospital Obrero, La Paz). The respective Institutional Review Boards waived the necessity for patient consent.

Note that full information on the approval of the study protocol must also be provided in the manuscript.

Field-specific reporting

Please select the one below that is the best fit for your research. If you are not sure, read the appropriate sections before making your selection.

☒ Life sciences ☐ Behavioural & social sciences ☐ Ecological, evolutionary & environmental sciences

For a reference copy of the document with all sections, see nature.com/documents/nr-reporting-summary-flat.pdf

Life sciences study design

All studies must disclose on these points even when the disclosure is negative.

Sample size	We sequenced 96 tumor and 34 normal samples from 35 acral melanoma patients, and additionally included 7 tumor and 2 normal samples from 2 publicly available patients. For each patient, we required two more tumor areas, primarily from multiple different progression stages (in situ, invasive and metastasis). Acral melanoma itself is a rare melanoma subtype, and cases with multiple progression stages available are difficult to obtain. Based on our prior experiences on studying the genetic evolution of cutaneous and uveal melanomas, as well as common practice in the field of tumor evolution, we determined that our sample size would be sufficient for our research purpose.
Data exclusions	No data or samples were excluded from the analysis
Replication	It is not a common practice to sequence multiple times for the same sample with the same sequencing approach, such as the exome-seq we

Replication	used in this study. However, results from exome-seq were replicated with different sequencing strategies, including targeted sequencing and whole-genome sequencing.
Randomization	Not relevant. Participants were all acral melanoma patients each with multiple tumor areas sequenced and uniformly analyzed.
Blinding	No blinding was performed for the data analysis of the study.

Reporting for specific materials, systems and methods

We require information from authors about some types of materials, experimental systems and methods used in many studies. Here, indicate whether each material, system or method listed is relevant to your study. If you are not sure if a list item applies to your research, read the appropriate section before selecting a response.

Materials & experimental systems

n/a	Involved in the study
☐	☒ Antibodies
☒	☐ Eukaryotic cell lines
☒	☐ Palaeontology and archaeology
☒	☐ Animals and other organisms
☒	☐ Clinical data
☒	☐ Dual use research of concern
☒	☐ Plants

Methods

n/a	Involved in the study
☒	☐ ChIP-seq
☒	☐ Flow cytometry
☒	☐ MRI-based neuroimaging

Antibodies

Antibodies used	YAP1 (12395S, Cell Signaling Technology), dilution 1:400. p16 (BSB-5828, Bio SB), ready-to-use.
Validation	These antibodies have been validated and routinely used for Immunohistochemistry in clinical setting at UCSF. Detailed webpages of the antibodies are: https://www.cellsignal.com/products/primary-antibodies/yap-1a12-mouse-mab/12395 and https://www.biosb.com/biosb-products/p16-antibody-mmab-16p04-jc12/

Plants

Seed stocks	N/A
Novel plant genotypes	N/A
Authentication	N/A
